# GCAS: An Integrated R Package and Shiny App for Comprehensive Cancer Data Analysis

**DOI:** 10.3390/biom16060823

**Published:** 2026-06-02

**Authors:** Jin Wang, Meidan Wei, Jiaxin Zhang, Xiangrong Song, Yaoyu Hu, Lexin Qin, Tingting Liang, Xinyu Zhu, Jianxiang Li

**Affiliations:** School of Public Health, Suzhou Medical College of Soochow University, Suzhou 215123, China; 20234247035@stu.suda.edu.cn (M.W.); 20244247010@stu.suda.edu.cn (J.Z.); 20234247003@stu.suda.edu.cn (X.S.); 20235247033@stu.suda.edu.cn (Y.H.); 2330506055@stu.suda.edu.cn (L.Q.); 2230412060@stu.suda.edu.cn (T.L.); 2330506091@stu.suda.edu.cn (X.Z.)

**Keywords:** bioinformatics tool, GEO database analysis, immune cell infiltration, anticancer drug sensitivity, cancer biomarker identification

## Abstract

Cancer research is pivotal for understanding cancer biology, discovering new therapeutic targets, and advancing precision medicine. However, it faces challenges such as data complexity, dispersed analytical tools, and the lack of a unified platform. To address these issues, we developed the GEO Cancer Analysis Suite (GCAS), an R package and visualization interface via shinyApp. GCAS includes four main modules: differential gene expression analysis, correlation studies, pan-cancer analysis, and immune infiltration and drug sensitivity analysis. These modules facilitate the identification of potential cancer biomarkers, elucidation of gene regulatory networks, comprehensive multi-cancer analysis, and assessment of gene expression in relation to immune cell infiltration and drug sensitivity. Using GCAS, *GAPDH* was found to be upregulated in multiple lung cancer and breast datasets and positively correlated with the m^6^A regulatory gene IGF2BP3. Further in vitro assays suggested that IGF2BP3 regulates *GAPDH* mRNA stability. Immune infiltration analysis indicated a negative correlation between *GAPDH* expression and CD4 T cell infiltration scores. Drug sensitivity analysis revealed a significant negative correlation between *GAPDH* expression and sensitivity to EGFR-targeting drugs, particularly Erlotinib. GCAS is a crucial tool in cancer research, simplifying data analysis and enhancing the discovery of novel biomarkers, immune landscape profiles, and drug sensitivity predictions, significantly contributing to cancer research and precision medicine.

## 1. Introduction

Cancer remains a leading cause of mortality worldwide and poses substantial challenges for researchers seeking to unravel its diverse biological mechanisms [1]. The advent of high-throughput sequencing technologies has generated vast amounts of genomic data, providing powerful opportunities to investigate tumor biology, patient heterogeneity, and therapeutic responses. The Gene Expression Omnibus (GEO, https://www.ncbi.nlm.nih.gov/geo/, accessed on 1 May 2025) is one of the most important repositories for such data, comprehensively collecting gene expression profiles across a wide range of cancer types [2,3]. However, effectively leveraging these datasets remains challenging, largely due to fragmented analytical tools and technical barriers associated with data retrieval, processing, and interpretation.

Several tools have been developed to facilitate GEO data analysis, including GEO2R and GEOquery. GEO2R is a user-friendly web-based utility provided by NCBI that enables researchers to compare two or more sample groups within GEO datasets and identify differentially expressed genes [4]. In contrast, GEOquery is an R package that allows direct programmatic access to GEO, making it easier to integrate GEO data into flexible, user-defined analysis pipelines in R [5]. Another widely used resource has been ONCOMINE (http://www.oncomine.org/, ceased operations on 17 January 2022), which aggregates data from GEO, TCGA, and published studies, representing one of the largest integrated databases and a powerful data-mining platform for cancer research [6]. However, as of January 2022, the ONCOMINE platform has ceased operation, leaving a notable gap in comprehensive, integrative tools for cancer genomics.

**Figure 1:**
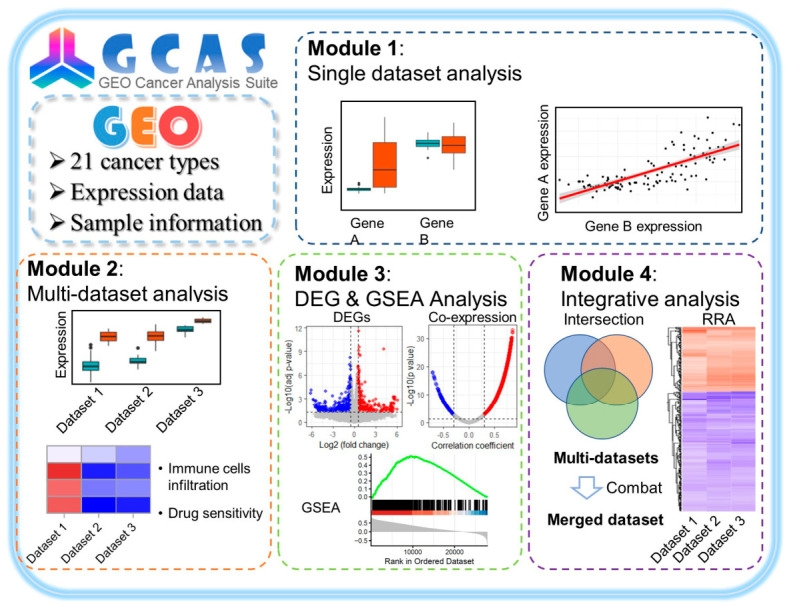
Overview of GCAS functions.

Despite these existing resources, there remains an unmet need for a unified, accessible platform that integrates multiple analytical methods for GEO-based cancer research. To address this gap, we introduce the GEO Cancer Analysis Suite (GCAS), a novel R package accompanied by an interactive visualization interface implemented via shinyApp (https://jingle.shinyapps.io/GCAS/, accessed on 1 June 2025). As illustrated in Figure 1, GCAS provides a streamlined, all-in-one solution for GEO data analysis, capable of handling diverse data types and analytical tasks. This tool enables researchers to seamlessly explore GEO datasets, perform integrative analyses across different cancer types, and derive actionable insights into cancer biology and precision medicine.

Glyceraldehyde-3-phosphate dehydrogenase (*GAPDH*) is a classical glycolytic enzyme that has long been used as a housekeeping gene for normalization in gene expression studies. However, *GAPDH* has been reported to be significantly overexpressed in multiple cancer types at both the RNA [7] and protein [8] levels, and accumulating evidence suggests that, beyond its canonical metabolic role, aberrant *GAPDH* expression is implicated in tumorigenesis and cancer progression [9]. Given these characteristics, we use *GAPDH* in this study as a representative example to demonstrate the application of GCAS in dissecting gene functions and elucidating their relevance to tumor biology.

## 2. Materials and Methods

### 2.1. Systematic Functional Comparison with Existing Tools

To clarify the positioning of GCAS relative to commonly used tools, we systematically compared data sources, analytical functionalities, working modes, extensibility, and reproducibility across GCAS, GEO2R, GEPIA2, UALCAN, and cBioPortal. As summarized in Table 1, GCAS is specifically designed as a GEO-centered, scriptable R package with an integrated Shiny interface, enabling local deployment and flexible pipeline integration, while providing built-in modules for differential expression, enrichment analysis, immune infiltration, co-expression/network analysis, drug-sensitivity prediction, and survival analysis.

### 2.2. Data Collection and Preprocessing

The GEO database was searched using various cancer names, with filter criteria set to species “Homo sapiens” and Number of Samples greater than or equal to 10, further excluding datasets that did not contain normal or adjacent non-tumor tissues. In our current database, we have included 21 tumor types: bladder cancer, lung cancer, breast cancer, colorectal cancer, prostate cancer, endometrial cancer, liver cancer, esophageal cancer, thyroid cancer, gastric cancer, glioma, head and neck cancer, cervical cancer, kidney cancer, skin cancer, ovarian cancer, pancreatic cancer, osteosarcoma, myeloma, leukemia, and lymphoma.

For microarray data, we downloaded the MINiML-formatted family file(s), which contain preprocessed and normalized expression data for each sample along with platform annotation information. Subsequently, data integration and probe annotation were performed using R, generating sample information. All probes were annotated with gene IDs, which were then converted to gene symbols.

### 2.3. Software and R Packages

This platform was fully developed using R software (Version 4.4.1), including the user interface, as well as analysis and visualization scripts. Table 2 lists the key packages and their main functions.

### 2.4. Immune Cell Infiltration Data

To comprehensively understand the tumor-immune interactions and their potential therapeutic implications, we analyzed immune cell infiltration scores for each tumor sample using the “IOBR (v2.2.3)” package in R [10]. This package synthesizes data from eight distinct algorithms—CIBERSORT (v0.1.0) [11], ESTIMATE (v1.0.13) [12], quanTIseq (v1.21.0) [13], TIMER (v1.2.0) [14], MCPCounter (v1.2.0) [15], xCell (v1.1.0) [16], and EPIC (v1.1.7) [17]—to provide a detailed assessment of the immune landscape within the tumor microenvironment. By integrating these diverse algorithms, we ensured a robust and multidimensional evaluation of immune cell infiltration across the samples.

### 2.5. Drug Sensitivity Analysis

To identify potential therapeutic vulnerabilities and understand the mechanisms of drug resistance, we analyzed the sensitivity of each tumor sample to a panel of 198 anticancer drugs using the “OncoPredict (v1.2.0)” package [18,19]. This analysis utilized IC50 data from tumor cell lines and high-throughput sequencing data from tumor samples available in the GDSC2.0 database. The drug sensitivity scores produced by OncoPredict are positively correlated with IC50 values, meaning that higher scores indicate greater resistance to the drugs. Furthermore, the 198 drugs were categorized based on their target pathways, including cell cycle regulation, WNT signaling, and P53 signaling pathways, as annotated in the GDSC2.0 database (https://www.cancerrxgene.org/, accessed on 8 July 2025). This pathway categorization provided insights into the potential mechanisms of drug action and resistance, enhancing our understanding of the complex interplay between drug efficacy and tumor biology.

### 2.6. Using the Analysis Tool

We provide two methods for users to utilize the Analysis tool:

Online Access via Shiny Application: Users can directly access the tool through a Shiny application deployed on the Shinyapps.io platform. The application can be accessed using the following link: https://jingle.shinyapps.io/GCAS/, accessed on 30 July 2025. By clicking this link, users can open and interact with the application in their web browser for immediate analysis.

Local Installation of GCAS (v0.2.0) R Package: Alternatively, users can install the GCAS R package locally in their R environment. This can be done by executing the following command in R: remotes::install_github(“WangJin93/GCAS”). After installation, the GCAS application can be launched locally with the command: GCAS::GCAS_app(). Additionally, the GCAS R package provides a broader array of functions for data acquisition, analysis, and visualization, which can be used directly within the R terminal, offering greater flexibility and functionality beyond the Shiny application.

### 2.7. CPTAC Analysis

The Clinical Proteomic Tumor Analysis Consortium (CPTAC) database provides a comprehensive resource for cancer proteomics. In this study, we focused on analyzing the expression of GAPDH in relation to IGF2BP3 using the ProteoCancer Analysis Suite (PCAS) [8]. Utilizing PCAS, we performed correlation analyses to assess the relationship between GAPDH and IGF2BP3 expression levels across the selected cancer types.

### 2.8. Cell Culture

A549 and H1299 lung cancer cell lines were obtained from the American Type Culture Collection (ATCC, Manassas, VA, USA). The cells were cultured in RPMI 1640 medium supplemented with 10% fetal bovine serum (FBS), 100 units/mL penicillin, and 100 µg/mL streptomycin. The cultures were maintained at 37 °C in a humidified incubator with 5% CO_2_. Cells were passaged at 70–80% confluency using 0.25% trypsin–EDTA solution and reseeded at an appropriate density for subsequent experiments.

### 2.9. IGF2BP3 Knockdown shRNA Plasmid Construction

To achieve *IGF2BP3* knockdown, specific shRNA sequences targeting *IGF2BP3* mRNA were designed using online tools and synthesized by a commercial vendor. The shRNA oligonucleotides were annealed and cloned into the pGreen-Puro shRNA vector (System Biosciences, Palo Alto, CA, USA) according to the manufacturer’s instructions. The recombinant plasmids were verified by DNA sequencing. Lipofectamine 2000 (Thermo Fisher Scientific, Waltham, MA, USA) was used for transfecting A549 and H1299 cells with the constructed plasmids, following the manufacturer’s protocol. Transfection efficiency was evaluated by fluorescence microscopy, and knockdown efficiency was confirmed by quantitative real-time PCR (qRT-PCR) and Western blot analysis.

### 2.10. RNA Stability Assay

The RNA stability assay was performed as previously described [20]. To evaluate *GAPDH* mRNA stability, cells were treated with 10 µg/mL actinomycin D (Sigma-Aldrich, St. Louis, MO, USA) and sampled at 0, 1, 2, 3, 4, 5, and 6 h. After extracting the total RNA, *GAPDH* mRNA decay was quantified by real-time PCR. The Ct values from different time points were normalized to the Ct value at t = 0 (ΔCt = Ct at each time point − Ct at t = 0). The relative RNA abundance at each time point was calculated using the formula 2^−ΔCt^.

### 2.11. Statistical Framework of GCAS

Differential expression analysis: GCAS compares gene expression between tumor and normal samples using parametric (Student’s *t*-test) or non-parametric (Wilcoxon rank-sum) tests. Multiple testing is controlled by the Benjamini–Hochberg (BH) method (default significance: adjusted *p* < 0.05). Output includes *p*-values, adjusted *p*-values, sample sizes, and standard volcano plots.

Correlation analysis: GCAS calculates Pearson or Spearman correlation coefficients to assess gene–gene associations. Results include correlation coefficient (r), *p*-value, BH-adjusted *p*-value, and 95% confidence intervals. Analyses can be restricted to tumor, normal, or all samples.

Multi-dataset integration and DEG in integrated data: For integration of multiple GEO datasets, GCAS applies ComBat to reduce batch effects while preserving biological variation. Differentially expressed genes in integrated data are identified using the limma framework, reporting log2 fold change and BH-adjusted *p*-values. Thresholds for log2 fold change and adjusted *p*-values are user-configurable and visualized with volcano plots.

### 2.12. Statistical Analysis

Data were analyzed using GraphPad V8.3.0. Student’s *t*-test was used for two-group comparisons, with *p* < 0.05 as the threshold for significance. RNA stability was analyzed using the one-phase decay model in GraphPad Prism, estimating mRNA half-life. Results are shown as mean ± SD, with *p* < 0.05 indicating significance.

## 3. Results

### 3.1. Demonstration of Module 1 “Single Gene Analysis”

**Figure 2:**
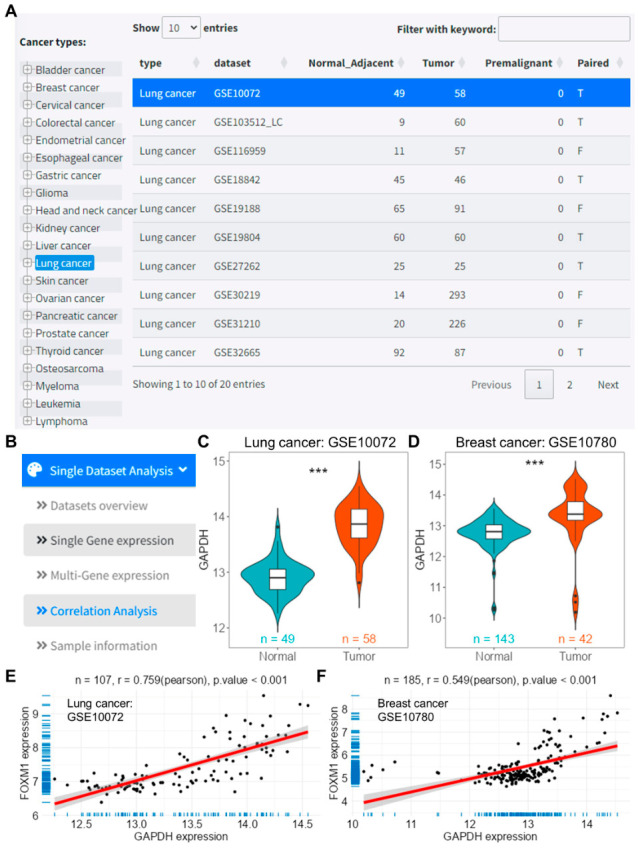
Demonstration of Module 1 functionalities: (**A**) Overview of datasets in this R package/shinyApp. (**B**) Overview of submodules in Module 1. Analysis of *GAPDH* expression in tumor and normal tissues using the “Single Gene Expression” submodule for the Lung cancer dataset GSE10072 (**C**) and the Breast cancer dataset GSE10780 (**D**). Scatter plots show the correlation analysis results between *GAPDH* and *FOXM1* expression using the “Correlation Analysis” submodule for the Lung cancer dataset GSE10072 (**E**) and the Breast cancer dataset GSE10780 (**F**). ***, *p* < 0.001 between two groups.

Figure 2A presents an overview of the datasets in this R package/shinyApp, which includes 21 types of tumors, comprising 228 independent studies and 23,177 samples in total. Figure 2B shows the overview of Module 1 functionalities, including “Single Gene Expression,” “Multi-gene Expression,” “Correlation Analysis,” and “Sample Information.” *GAPDH* was found to be significantly overexpressed in tumor tissues based on the Lung cancer dataset GSE10072 (Figure 2C) and the Breast cancer dataset GSE10780 (Figure 2D). Correlation analysis results indicated a significant positive correlation between *GAPDH* and *FOXM1* expression in the GSE10072 (Figure 2E) and GSE10780 (Figure 2F) datasets (R = 0.759 and 0.549, respectively, both *p* < 0.05).

### 3.2. GAPDH Expression and Regulatory Mechanisms in Lung Cancer Based on Module 2

**Figure 3:**
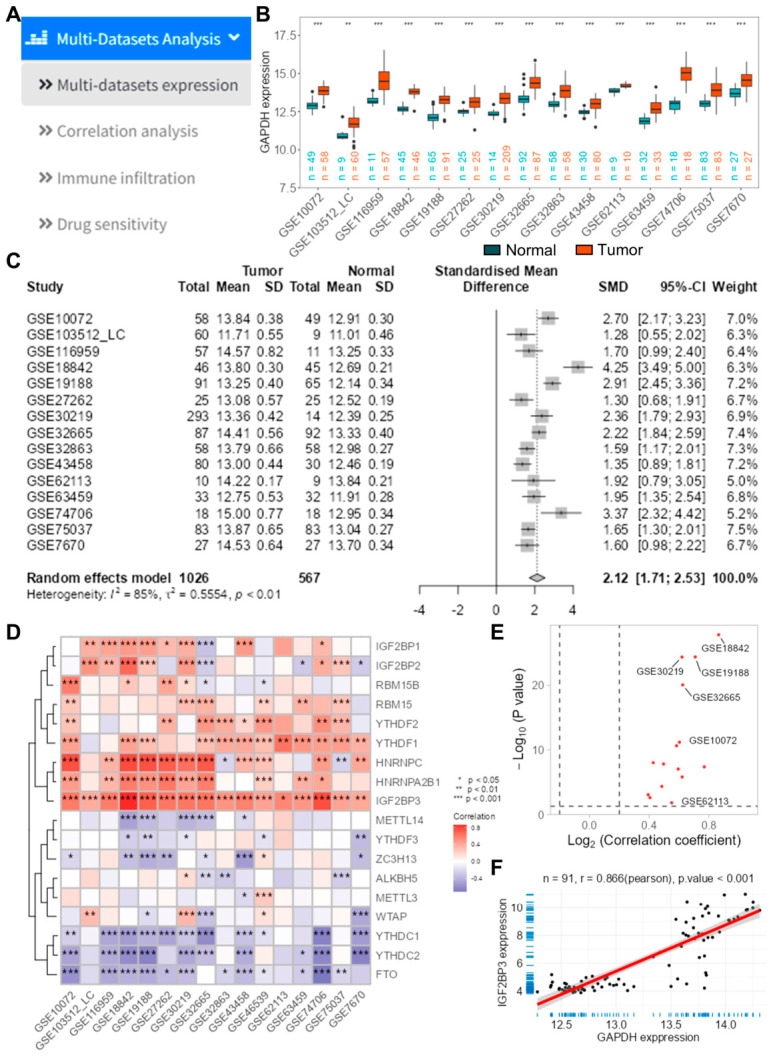
Analysis of *GAPDH* expression and correlation with m^6^A regulatory gene expression in lung cancer using the “Multi-dataset Expression” and “Correlation Analysis” submodules of Module 2: (**A**) Overview of submodules in Module 2. (**B**) Analysis of *GAPDH* expression differences between tumor and normal tissues in multiple lung cancer datasets using the “Multi-dataset Expression” submodule. **, *p* < 0.01 between two groups. ***, *p* < 0.001 between two groups. (**C**) Forest plot showing meta-analysis of *GAPDH* expression differences between tumor and normal tissues across multiple datasets. (**D**) Heatmap showing correlation analysis results between *GAPDH* expression and m^6^A regulatory gene expression across multiple lung cancer datasets using the “Correlation Analysis” submodule. (**E**) Scatter plot showing correlation analysis results between *GAPDH* and *IGF2BP3* expression across multiple lung cancer datasets. (**F**) Scatter plot showing correlation analysis results between *GAPDH* and *IGF2BP3* expression in the GSE18842 dataset.

Module 2 is designed for expression and correlation analysis across multiple datasets, encompassing four submodules: “Multi-dataset Expression,” “Correlation Analysis,” “Immune Infiltration,” and “Drug Sensitivity” (Figure 3A). Using the “Multi-dataset Expression” submodule, we found that *GAPDH* is significantly overexpressed in multiple lung cancer datasets (all *p* < 0.05, Figure 3B). This module provides general statistical results for tumor and normal samples in each dataset (Supplementary Material). Furthermore, this module offers meta-analysis and visualization of gene expression across multiple datasets. The results indicate that *GAPDH* is upregulated in multiple datasets. Due to significant heterogeneity, a random-effects model was applied (Figure 3C, SMD = 2.12, *p* < 0.01). Using the “Correlation Analysis” submodule, we examined the correlation between *GAPDH* expression and m^6^A regulatory genes across multiple lung cancer datasets. Results showed significant correlations with several m^6^A regulatory genes (Figure 3D), particularly IGF2BP3, which was significantly positively correlated with *GAPDH* expression in all datasets (Figure 3E, r > 0.2, *p* < 0.05), with the highest correlation observed in the GSE18842 dataset (Figure 3F, r = 0.866). Similarly, we analyzed multiple breast cancer datasets and found that *GAPDH* was significantly upregulated in tumor tissues (*p* < 0.05, Figure S1A,B). The expression of m^6^A regulatory genes was also significantly correlated with *GAPDH* expression, especially *IGF2BP3* (Figure S1C).

### 3.3. IGF2BP3 Regulates GAPDH mRNA Stability

**Figure 4:**
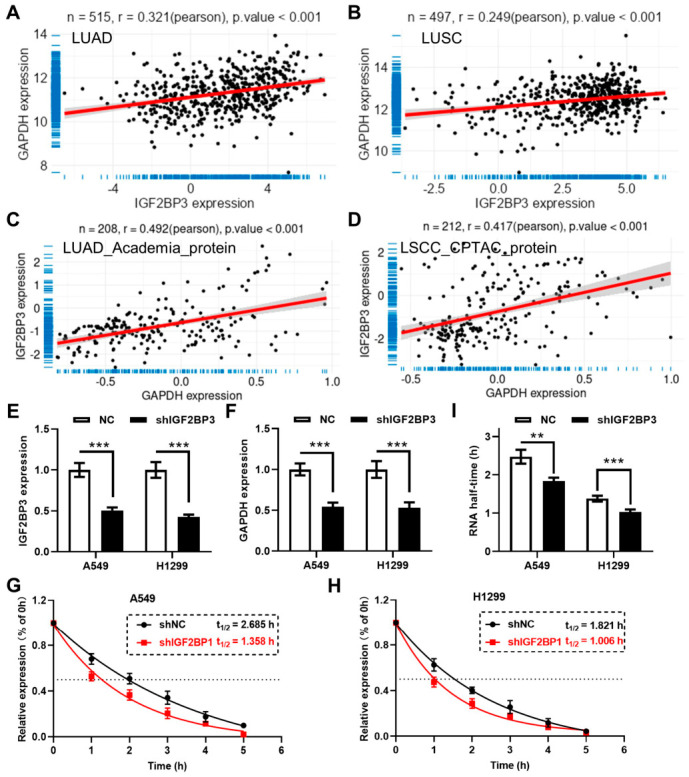
In vitro validation of *IGF2BP3* regulation of *GAPDH*. Scatter plots show the correlation between *IGF2BP3* mRNA expression and *GAPDH* expression in the TCGA LUAD (**A**) and LUSC (**B**) datasets. Scatter plots show the correlation between IGF2BP3 protein expression and GAPDH expression in the CPTAC LUAD (**C**) and LUSC (**D**) datasets. qPCR analysis of *IGF2BP3* (**E**) and *GAPDH* (**F**) expression changes following *IGF2BP3* knockdown in lung cancer cells. Degradation curves of *GAPDH* mRNA in A549 (**G**) and H1299 (**H**) cells following actinomycin D treatment and *IGF2BP3* knockdown. (**I**) Calculation of *GAPDH* half-life in A549 and H1299 cells post-*IGF2BP3* knockdown based on RNA degradation curves. TCGA: The Cancer Genome Atlas. LUAD: Lung Adenocarcinoma. LUSC: Lung Squamous Cell Carcinoma. CPTAC: Clinical Proteomic Tumor Analysis Consortium. **, *p* < 0.01 between two groups. ***, *p* < 0.001 between two groups.

To validate the analytical reliability of our developed GCAS platform, we first analyzed the correlation between *IGF2BP3* and *GAPDH* regulation based on the TCGA and CPTAC databases. We observed a significant positive correlation between *IGF2BP3* mRNA expression and *GAPDH* expression in the LUAD (Figure 4A) and LUSC (Figure 4B) datasets. Furthermore, analysis of the CPTAC database also showed a significant positive correlation between IGF2BP3 protein expression and GAPDH expression in LUAD (Figure 4C) and LUSC (Figure 4D) datasets. qPCR results further demonstrated that knockdown of *IGF2BP3* in lung cancer cells led to a significant decrease in the expression of both *IGF2BP3* (Figure 4E) and *GAPDH* (Figure 4F). To explore changes in *GAPDH* mRNA stability, we conducted actinomycin D treatment experiments. The results showed that knockdown of *IGF2BP3* accelerated the degradation of *GAPDH* mRNA in A549 (Figure 4G) and H1299 (Figure 4H) cells, significantly shortening its half-life (Figure 4I).

### 3.4. Analysis of GAPDH Correlation with Immune Cell Infiltration and Drug Sensitivity Using Module 2

**Figure 5:**
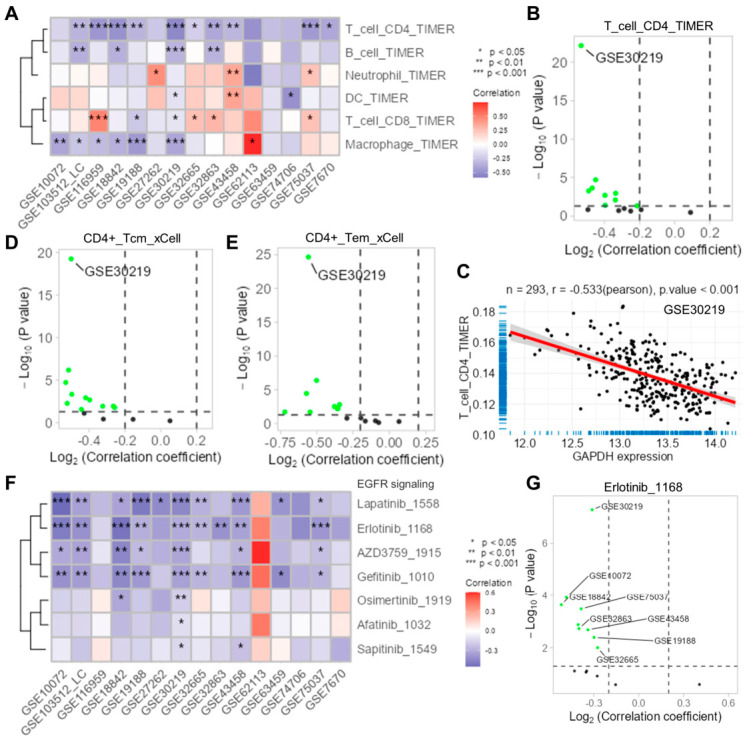
Correlation analysis of *GAPDH* with immune cell infiltration and antitumor drug sensitivity in lung cancer using the “Immune infiltration” and “Drug sensitivity” submodules of Module 2: (**A**) Heatmap showing the correlation between *GAPDH* expression and immune cell infiltration scores derived from the TIMER algorithm across multiple lung cancer datasets. (**B**) Scatter plot showing the correlation between *GAPDH* expression and CD4 T cell infiltration scores derived from the TIMER algorithm in multiple lung cancer datasets. (**C**) Scatter plot showing the correlation between *GAPDH* expression and CD4 T cell infiltration scores derived from the TIMER algorithm in the GSE30219 dataset. Scatter plots showing the correlation between *GAPDH* expression and CD4 Tcm (**D**) and CD4 Tem (**E**) cell infiltration scores derived from the xCell algorithm in multiple lung cancer datasets. (**F**) Heatmap showing the correlation between *GAPDH* expression and sensitivity scores for drugs targeting the EGFR pathway in multiple lung cancer datasets. (**G**) Scatter plot showing the correlation between *GAPDH* expression and Erlotinib sensitivity scores in multiple lung cancer datasets. Tcm: central memory T cells. Tem: effector memory T cells.

To further investigate the function of *GAPDH* in lung cancer, we utilized the “Immune infiltration” and “Drug sensitivity” submodules of Module 2 to analyze the correlation between *GAPDH* expression and immune cell infiltration as well as antitumor drug sensitivity. As shown in the heatmap in Figure 5A, *GAPDH* expression is significantly correlated with immune cell infiltration scores derived from the TIMER algorithm across multiple lung cancer datasets. Specifically, *GAPDH* expression exhibited a consistent negative correlation with CD4 T cell infiltration scores in multiple datasets (Figure 5B), with the lowest correlation observed in the GSE30219 dataset (r = −0.533, Figure 5C). Further analysis using the xCell algorithm also showed a significant correlation between *GAPDH* and most immune cell infiltration scores (Figure S2). Notably, *GAPDH* expression was significantly negatively correlated with CD4 Tcm (Figure 5D) and CD4 Tem (Figure 5E) cell infiltration scores across multiple lung cancer datasets. Regarding antitumor drug sensitivity, *GAPDH* expression was significantly correlated with sensitivity scores for drugs targeting the EGFR pathway in multiple lung cancer datasets (Figure 5F), with a particularly notable negative correlation with Erlotinib sensitivity scores (Figure 5G). Additionally, correlation analysis revealed a significant negative correlation between *GAPDH* expression and sensitivity to various cell cycle-related drugs across multiple datasets (Figure S3).

### 3.5. Differential Gene Expression and Enrichment Analysis Based on Module 3

**Figure 6:**
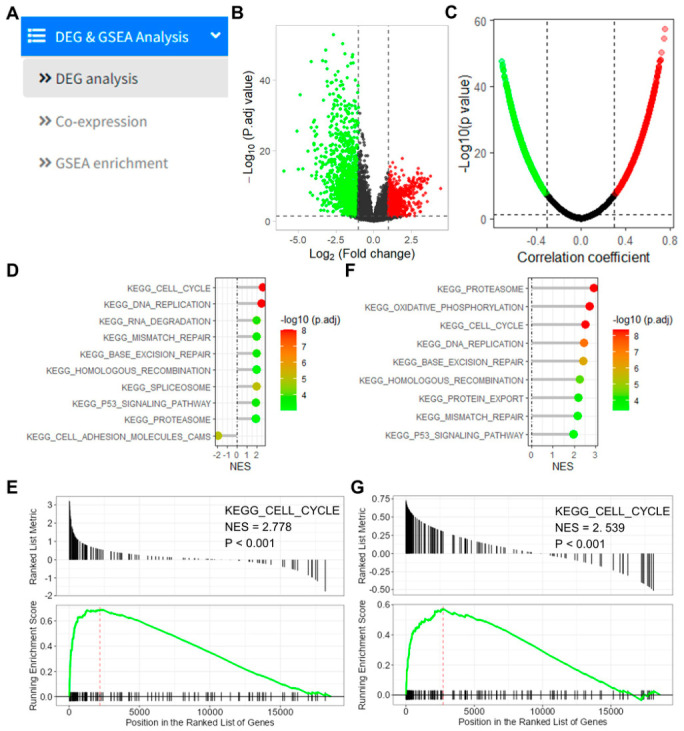
Differential gene expression analysis and *GAPDH* single-gene GSEA enrichment analysis using Module 3 on the GSE30219 dataset: (**A**) Overview of submodules in Module 3. (**B**) Volcano plot showing differentially expressed genes between tumor and normal samples in the GSE30219 dataset using the “DEG analysis” submodule. (**C**) Volcano plot showing genes co-expressed with *GAPDH* in the GSE30219 dataset using the “Co-expression” submodule. (**D**) Lollipop chart displaying KEGG pathway enrichment of differentially expressed genes using the “GSEA enrichment” submodule. (**E**) GSEA plot showing significant enrichment of differentially expressed genes in the cell cycle pathway. (**F**) Lollipop chart displaying KEGG pathway enrichment of *GAPDH* co-expressed genes. (**G**) GSEA plot showing significant enrichment of co-expressed genes in the cell cycle pathway. GSEA: gene set enrichment analysis. DEG: differentially expressed gene. KEGG: Kyoto encyclopedia of genes and genomes.

This module aims to perform differential gene expression analysis and co-expression analysis of specific genes in the dataset, followed by GSEA enrichment analysis based on the results. Figure 6A provides an overview of the submodules within Module 3. Using the GSE30219 dataset as an example, the “DEG analysis” submodule identified differentially expressed genes between tumor and normal samples, visualized in a volcano plot (Figure 6B). Subsequently, the “Co-expression” submodule analyzed genes co-expressed with *GAPDH* in the GSE30219 dataset, also presented in a volcano plot (Figure 6C). Next, the “GSEA enrichment” submodule performed KEGG pathway enrichment analysis on the differentially expressed genes, depicted in a lollipop chart, revealing significant enrichment in multiple KEGG pathways (Figure 6D), with the cell cycle pathway showing the highest NES (NES = 2.778, Figure 6E). Similarly, enrichment analysis of *GAPDH* co-expressed genes was conducted, with the results visualized in a lollipop chart, highlighting significantly enriched pathways (Figure 6F), including the cell cycle (NES = 2.539, Figure 6G).

### 3.6. Integrated Analysis of Multiple Datasets Based on Module 4

**Figure 7:**
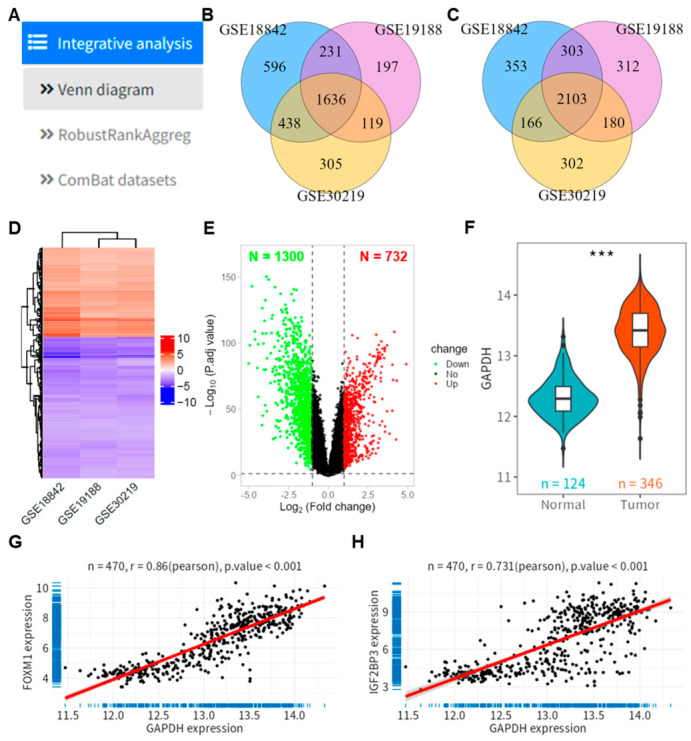
Integrated analysis of multiple datasets using GCAS: (**A**) Overview of submodules in Module 4. Visualization of intersections of upregulated (**B**) and downregulated (**C**) differentially expressed genes in GSE19188, GSE30219, and GSE18842 using the “Venn diagram” submodule. (**D**) Integration of differentially expressed genes across the three datasets using the RobustRankAggreg algorithm. (**E**) Volcano plot showing differentially expressed genes in the merged dataset after using the “ComBat datasets” submodule. (**F**) Violin plot showing the *GAPDH* expression in the merged dataset. Scatter plots showing correlations between the expression of *GAPDH* and *FOXM1* (**G**) and *IGF2BP3* (**H**) in the merged dataset. GCAS, GEO Cancer Analysis Suite. ***, *p* < 0.001 between two groups.

Module 4 is crucial for integrating and visualizing differentially expressed genes and merging datasets. Figure 7A provides an overview of the submodules within Module 4. Using three lung cancer datasets—GSE19188, GSE30219, and GSE18842—as examples, differential gene expression analysis was first performed using Module 3. The results were then imported into the “Venn diagram” submodule, which visualized the intersections of upregulated (Figure 7B) and downregulated (Figure 7C) genes. Next, the RobustRankAggreg algorithm was applied to integrate differentially expressed genes across the three datasets, revealing consistent differentially expressed genes (Figure 7D). To further enhance the robustness of the analysis, the “ComBat datasets” submodule was used to merge the three datasets, followed by differential gene expression analysis on the merged dataset, visualized in a volcano plot (Figure 7E). Also, the *GAPDH* expression value was extracted from the merged dataset, and the tumor samples show a higher expression than normal samples (Figure 7F). The further correlation analysis validated the negative correlations between the expression of *GAPDH* and *FOXM1* (Figure 7G) and *IGF2BP3* (Figure 7H) in the merged dataset.

## 4. Discussion

Malignant tumors remain a major cause of premature mortality worldwide, highlighting the need for comprehensive analytical tools based on large-scale cancer datasets. The Gene Cancer Analysis Suite GCAS addresses this need by integrating an R package and Shiny application with multiple high-throughput analysis modules. A major strength of GCAS is its broad dataset coverage, including 228 independent GEO studies across 21 cancer types and 23,177 samples. This resource improves access to public cancer transcriptomic data and enhances the robustness and generalizability of downstream analyses. GCAS adopts a modular design that allows users to build flexible workflows according to specific research aims. It supports single-gene and multi-gene analyses, differential expression analysis, multi-dataset integration, immune infiltration analysis, and drug sensitivity evaluation. These functions enable systematic assessment of gene expression patterns and their biological or clinical relevance in cancer. The embedded visualization tools in the Shiny application further facilitate interpretation and presentation of complex results.

Multi-dataset integration is a central feature of GCAS. By combining independent studies, GCAS increases statistical power, reduces dataset-specific bias, and improves the reliability of molecular findings. For differential analysis, GCAS provides intersection-based integration and the RobustRankAggreg algorithm, which identifies consistently ranked features across datasets [21]. For expression matrix integration, GCAS applies the ComBat function from the sva package to reduce batch effects while preserving biological variation [22]. These strategies support more reliable and interpretable multi-cohort cancer analyses.

*GAPDH* exhibits significant overexpression in various cancers, a phenomenon extensively documented in our previous studies at both RNA [7] and protein [8] levels. This aberrant expression suggests its potential role in tumorigenesis and progression. Our research indicates that *GAPDH* is involved not only in cancer cell metabolism but also in the tumor immune microenvironment and drug sensitivity. T cell exhaustion has become a recognized pattern of T cell dysfunction in cancer [23,24]. The negative correlation between *GAPDH* expression and CD4+ T cell infiltration scores suggests its potential role in modulating the tumor immune environment, providing clues for further investigation into *GAPDH*’s involvement in immune evasion mechanisms. Additionally, the significant association of *GAPDH* expression with sensitivity to various anticancer drugs indicates that *GAPDH* could serve as a predictive biomarker for drug response, guiding therapeutic strategies.

Regarding the regulatory mechanisms of GAPDH, previous studies have shown that its aberrant expression in tumors may be influenced by multiple layers of regulation, including DNA methylation, copy number variation, and transcriptional activation by FOXM1 [7]. Consistently, our tool also identified a significant expression correlation between FOXM1 and GAPDH, further supporting the potential contribution of transcriptional regulation to GAPDH dysregulation in cancer. In addition to transcriptional and genomic regulation, emerging evidence indicates that post-transcriptional mechanisms, particularly N6-methyladenosine m^6^A modification, play an essential role in controlling mRNA fate, including mRNA stability, translation efficiency, and degradation [25,26]. Previous studies based on the CPTAC database and GCAS analysis tools suggested that GAPDH may be regulated by m^6^A modification and the m^6^A reader protein IGF2BP1 [8]. In the present study, we further validated the correlation between GAPDH expression and m^6^A regulatory genes across multiple independent datasets and identified IGF2BP3 as another potential reader protein associated with GAPDH regulation.

IGF2BP3 belongs to the IGF2BP family of m^6^A reader proteins, which recognize m^6^A-modified transcripts and enhance the stability and translation of target mRNAs [27,28]. Notably, IGF2BP3 has been widely implicated in tumor progression, metastasis, stemness maintenance, and poor prognosis in multiple cancer types [29,30]. Our experimental validation further suggested that IGF2BP3 may increase GAPDH expression by maintaining GAPDH mRNA stability. These findings indicate that GAPDH upregulation in tumors may not only result from genomic or transcriptional alterations but also from m^6^A-dependent post-transcriptional regulation. This regulatory axis highlights the importance of epitranscriptomic control in cancer metabolism and provides new insight into the functional contribution of GAPDH to tumorigenesis.

## 5. Conclusions

The present work is primarily a method-oriented study aimed at introducing GCAS as an integrative, GEO-centered cancer transcriptomic analysis platform and illustrating its ability to generate biologically plausible, literature-consistent hypotheses, rather than providing exhaustive mechanistic proof for any single gene or pathway. Within this scope, GCAS demonstrates substantial potential for future cancer research applications.

Looking ahead, GCAS can be further expanded by incorporating additional datasets that cover a broader range of cancer types and by integrating other high-throughput data modalities, such as single-cell transcriptomics and multi-omics data. Extending its analytical repertoire to include genomic variation (e.g., somatic mutations, copy-number alterations) and epigenetic profiling will further enhance its utility for comprehensive cancer genomics studies. In the context of precision medicine, GCAS can be applied to patient-specific gene expression profiles as a hypothesis-generating tool to support the design of personalized therapeutic strategies. Moreover, by providing both a scriptable R package and a user-friendly Shiny interface, GCAS is positioned as a shared research platform that can facilitate interdisciplinary collaboration among bioinformaticians, molecular biologists, and clinicians, thereby helping to drive continued advances in cancer research.

## Figures and Tables

**Table 1:** Functional comparison between GCAS and representative cancer genomics analysis tools.

Dimension	GCAS	GEO2R	GEPIA2	UALCAN	cBioPortal
Data sources	GEO	GEO	TCGA, GTEx	TCGA	TCGA, ICGC, and other published cohorts
Access mode/working mode	R package + Shiny web interface; can run locally or online	Web interface only	Web interface only	Web interface only	Web interface (plus API/data download)
Multi-dataset/integrative analysis	Yes (intersection, RRA and ConBat)	No	Limited	No	Limited
Differential expression analysis	Yes (multiple GEO datasets; custom contrasts)	Yes (within a single GEO dataset)	Yes (tumor vs. normal or group comparisons)	Yes (tumor vs. normal; subgroup comparisons)	Yes (through built-in analysis modules)
GSEA and pathway analysis	Yes	No	Limited/indirect	Limited	Limited/indirect
Immune infiltration analysis	Yes (integrated immune cell infiltration estimation and visualization)	No	Limited (some immune-related functions)	Limited (some immune-related analyses, depending on version)	Limited (depends on specific study/module; not core)
Co-expression	Yes	No	Limited	Limited	Limited

**Table 2:** R packages used in the GCAS platform.

R Package	Functionality Description
Shiny (v1.11.1)	Builds the interactive web interface (Shiny app).
bs4Dash (v2.3.5)	Provides the dashboard layout and visual theme for the Shiny app.
shinyWidgets (v0.9.0)	Adds enhanced UI components (e.g., advanced buttons, sliders).
ggplot2 (v4.0.0)	Generates publication-quality plots and visualizations.
ggpubr (v0.6.3)	Supports statistical plotting (e.g., group comparisons, boxplots).
dplyr (v1.1.4)	Performs data manipulation and preprocessing.
RMySQL (v0.11.3)	Connects to and queries MySQL databases (data retrieval).
limma (v3.58.1)	Identifies differentially expressed genes from expression data.
Psych (v2.5.6)	Conducts correlation analyses (e.g., via corr.test).
IOBR (v2.2.3)	Performs immune-related and immune–oncology analyses.
oncoPredict (v1.2.0)	Predicts drug response based on gene expression profiles.
clusterProfiler (v4.20.0)	Conducts functional enrichment analysis (GO, KEGG, etc.).
Sva (v3.20.0)	Removes batch effects (e.g., ComBat) in multi-dataset integration.
VennDiagram (v1.8.2)	Draws Venn diagrams to visualize overlaps of gene sets.
RobustRankAggreg (v1.2.1)	Integrates multiple ranked gene lists using robust rank aggregation (RRA).

## Data Availability

The datasets analyzed for this study are publicly available in the Gene Expression Omnibus (GEO, https://www.ncbi.nlm.nih.gov/geo/, accessed on 1 May 2025). The PCAS package source code has been published on GitHub: https://github.com/WangJin93/PCAS, accessed on 1 May 2025.

## Supplementary Materials

The following supporting information can be downloaded at: https://www.mdpi.com/article/10.3390/biom16060823/s1. Figure S1: Analysis of *GAPDH* expression and correlation with m^6^A regulatory gene expression in breast cancer using Module 2; Figure S2: Heatmap showing the correlation between *GAPDH* expression and immune cell infiltration scores derived from the xCell algorithm across multiple lung cancer datasets; Figure S3: Correlation analysis of *GAPDH* with the sensitivity of antitumor drug targeting cell cycle process in lung cancer.

## Abbreviations

The following abbreviations are used in this manuscript:

GCAS	GEO Cancer Analysis Suite
GEO	Gene Expression Omnibus
TCGA	The Cancer Genome Atlas
CPTAC	Clinical Proteomic Tumor Analysis Consortium
LUAD	Lung Adenocarcinoma
LUSC	Lung Squamous Cell Carcinoma
PPP	Pentose Phosphate Pathway
TIMER	Tumor Immune Estimation Resource
GSEA	Gene Set Enrichment Analysis
DEGs	Differentially Expressed Genes
ROS	Reactive Oxygen Species
ATP	Adenosine 5′-Triphosphate
GTEx	Genotype-Tissue Expression
TFTF	TF-Target Finder
PCAS	ProteoCancer Analysis Suite
SMD	Standardized Mean Difference
IC50	Half Maximal Inhibitory Concentration
qPCR	Quantitative Polymerase Chain Reaction
m^6^A	N6-methyladenosine
mRNA	Messenger RNA
shRNA	Short Hairpin RNA
KEGG	Kyoto Encyclopedia of Genes and Genomes
NES	Normalized Enrichment Score
EdU	5-Ethynyl-2′-Deoxyuridine
