# Supplementary material for: GCAS: An Integrated R Package and Shiny App for Comprehensive Cancer Data Analysis
Source: Biomolecules. 2026 Jun 2;16(6):823. doi: 10.3390/biom16060823 (PMC13296616; doi:10.3390/biom16060823)
Supplement: Supplementary file 1 [file biomolecules-16-00823-s001.zip › biomolecules-4292919-supplementary.pdf]

Supporting Information

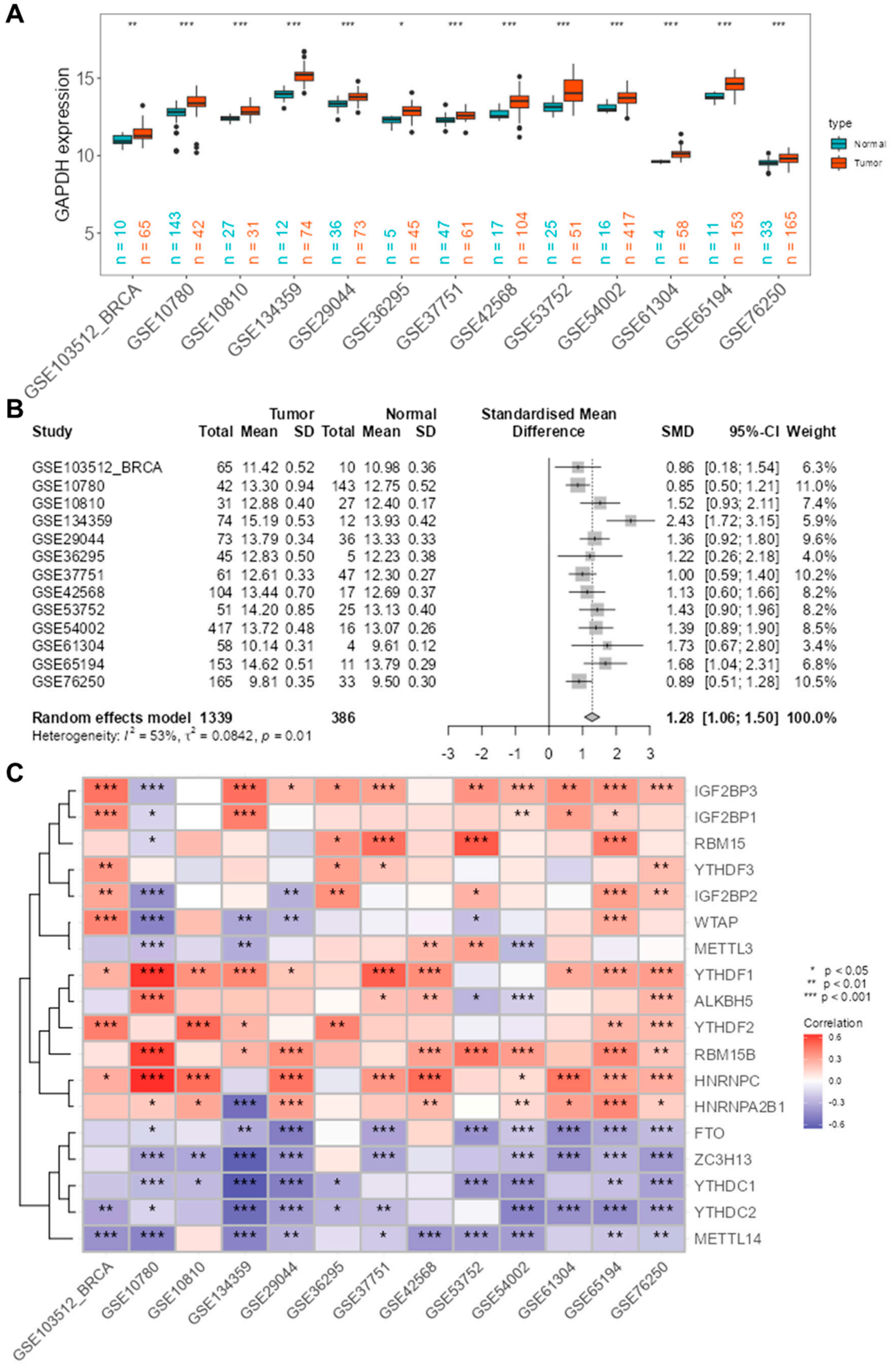

Figure S1. Analysis of GAPDH expression and correlation with m^6A regulatory gene expression in breast cancer using Module 2. (A) Analysis of GAPDH expression differences between tumor and normal tissues in multiple breast cancer datasets using the "Multi-datasets Expression" submodule. (B) Forest plot showing meta-analysis of GAPDH expression differences between tumor and normal tissues across multiple datasets. (C) Heatmap showing correlation analysis results between GAPDH expression and m^6A regulatory gene expression across multiple breast cancer datasets using the "Correlation Analysis" submodule.

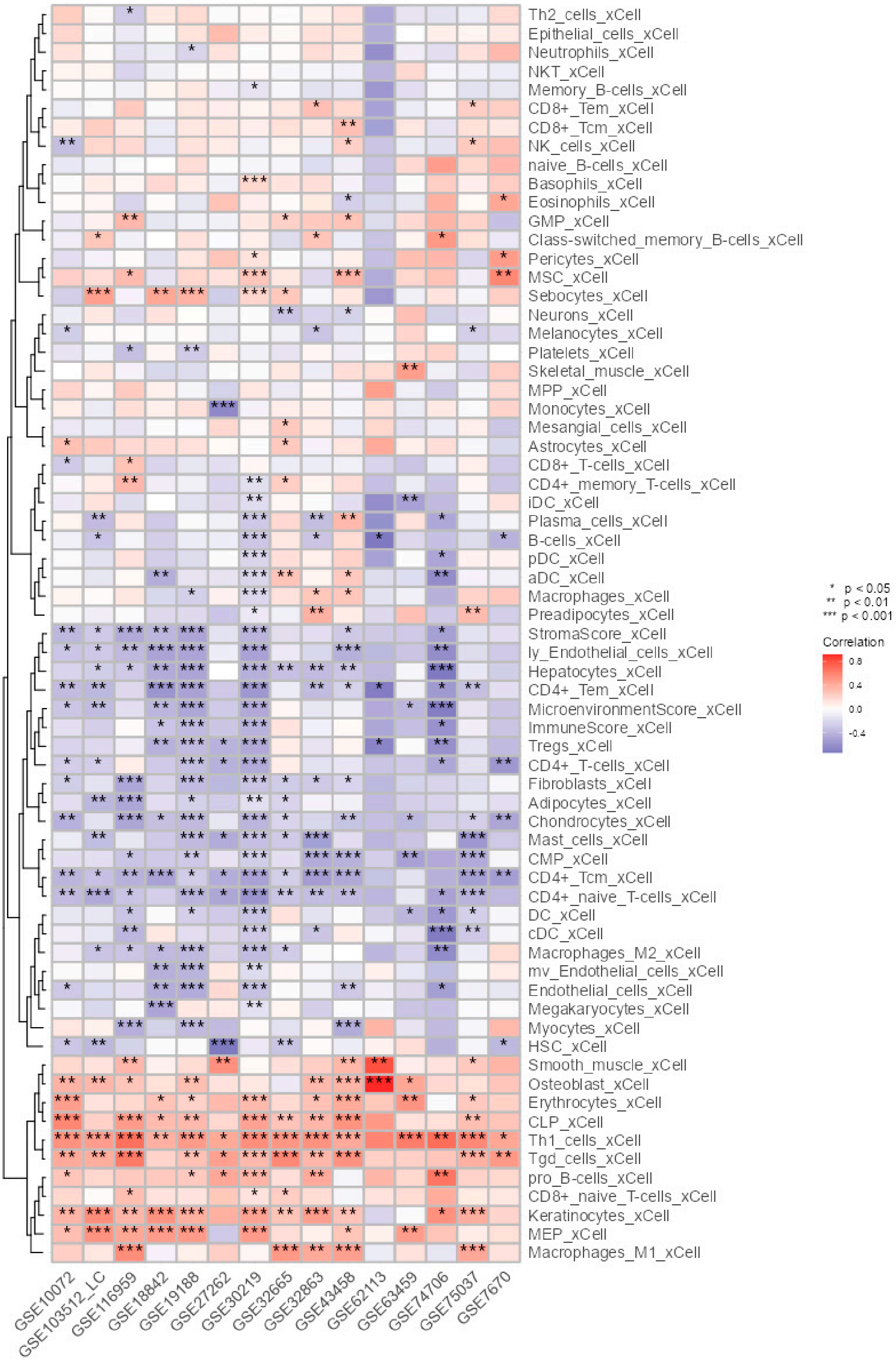

Figure S2. Heatmap showing the correlation between GAPDH expression and immune cell infiltration scores derived from the xCell algorithm across multiple lung cancer datasets.

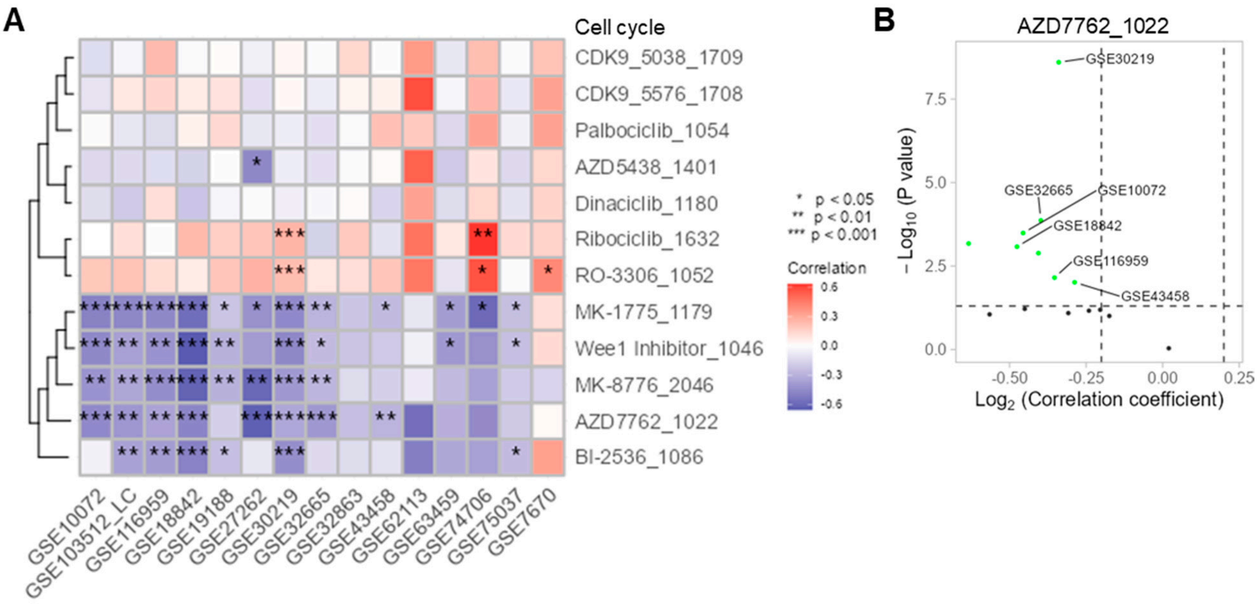

Figure S3. Correlation analysis of GAPDH with the sensitivity of antitumor drug targeting cell cycle process in lung cancer. (A) Heatmap showing the correlation between GAPDH expression and sensitivity scores for drugs targeting cell cycle in multiple lung cancer datasets. (B) Scatter plot showing the correlation between GAPDH expression and AZD7762 sensitivity scores in multiple lung cancer datasets.
